# One Strain Many Compounds Approach for Anti-*Trypanosoma cruzi* Compounds: Empowering the Marine Bacterium *Metabacillus indicus*

**DOI:** 10.1021/acsomega.4c10784

**Published:** 2025-05-01

**Authors:** Beatriz A. Andrade, Augusto L. dos Santos, Dayana A. S. Ferreira, Mariana B. Abiuzi, Daniel P. Vieira, Marina M. Gonçalves, João Henrique G. Lago, Patricia Sartorelli, Andre G. Tempone

**Affiliations:** † Pathophysiology Laboratory, Instituto Butantan, Av. Vital Brazil, 1500, São Paulo, SP 05503-900, Brazil; ‡ Postgraduate Program in Infectious Diseases and Global Health - Infectious and Parasitic Diseases, University of São Paulo School of Medicine, Av. Dr. Enéas Carvalho de Aguiar, 470, São Paulo 05403 000, Brazil; § Instituto de Ciências Ambientais, Químicas e Farmacêuticas, Universidade Federal de São Paulo (UNIFESP), Diadema Campus, São Paulo, SP 09913-030, Brazil; ∥ Lab de Radiobiologia do Centro de Biotecnologia - CEBIO (IPEN-CNEN/SP), Av. Lineu Prestes 2242, Cidade Universitária, São Paulo, SP 05508-000, Brazil; ⊥ Centre of Natural Sciences and Humanities, Universidade Federal do ABC, Sao Paulo 09210-580, Brazil

## Abstract

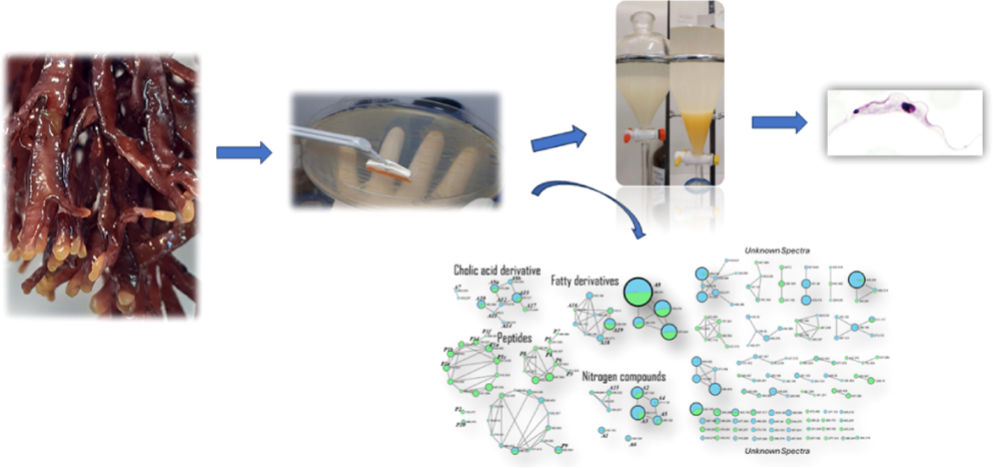

Neglected tropical diseases as Chagas disease (CD) affect more than eight million people, mainly in the Americas, causing fatal cardiovascular outcomes. Relying on two old, toxic, and low efficacy drugs for treatment, there is an urgent need for new candidates. Comprising a high chemodiversity, marine bacteria are a rich source of small molecules with potential against human pathogens. Cultivation-based strategies of bacteria, such as the one strain many compounds (OSMAC) approach, have proven to be a simple and promising tool for drug discovery, with the ability to stimulate the expression of cryptic genes in microorganisms. In this study, using the OSMAC, we evaluated the potential of the marine bacteria *Metabacillus indicus* to produce anti-*Trypanosoma cruzi* compounds with higher potency. The *M. indicus* was cultivated under different conditions, subdivided into four groups, as nutritional, physical, biological, and chemical alterations. For comparisons, the extract obtained from the bacteria in Marine Broth (static) at 25 °C was used as a control and resulted in an EC_50_ value of 28 μg/mL against the trypomastigotes. The physical alterations proved to be the most effective approach to improve the potency of *M. indicus* metabolites, resulting in EC_50_ values between 3 and 26 μg/mL. The cultivation in Marine Agar potentiated the antitrypanosomal metabolites by 8.4-fold. When exposed to cobalt-60 γ radiation (0.5 kGy), the bacteria produced metabolites with 2-fold higher antitrypanosomal potency. The nutritional alterations resulted in potent metabolites, with EC_50_ values between 11 and 18 μg/mL, while biological alterations resulted in EC_50_ values between 11 and 28 μg/mL. Addition of *T. cruzi* and *Leishmania infantum* antigens and co-cultivation with *Acinetobacter baumannii*, enhanced by 2-fold the potency. Chemical elicitors such as DMSO and EtOH demonstrated no improvements for *M. indicus* cultivation. The chemical profile of *M. indicus* was analyzed using NMR and UHPLC-ESI-HR-MS/MS and processed using the GNPS platform, which led to the annotation of nucleosides, dipeptides, steroids, and fatty acid derivatives. These findings confirmed that the OSMAC approach yielded not only distinct antitrypanosomal activities but also distinct metabolomic profiles in *M. indicus* that could be exploited for drug discovery studies for Chagas disease.

## 1 Introduction

Transmitted by triatomine insects, Chagas disease or American trypanosomiasis is an infectious disease caused by the protozoan *Trypanosoma cruzi*.^1^ This disease affects more than eight million people worldwide and is included in the neglected tropical diseases (NTDs) list of the World Health Organization (WHO).^2^ The Americas are regions most affected by the CD which is endemic in 21 Latin American countries, with an annual incidence of 28,000 cases and 12,000 deaths.^3^ In recent decades, globalization has brought a new dimension to CD. The disease, which was often associated with rural areas, has now been diagnosed in urban areas. Human migration, both regional and international, has allowed CD to spread from rural to urban areas in endemic countries, and also from endemic countries to other countries around the world.^4^ The most affected countries outside Latin America are the United States, Canada, Europe, Australia, and Japan, which have approximately 26 million residents in Latin America and around 400,000 individuals infected with CD.^4,5^ Since the early 1970s, only two drugs have been approved for CD in Brazil, benznidazole and nifurtimox.^6,7^ However, both drugs require long periods of treatment and can cause various adverse effects, including allergies, dermatitis, pruritus, and gastrointestinal intolerance.^8,9^ Studies published in recent years show that benznidazole can decrease the parasites in the bloodstream, but it is inefficient to reduce the cardiac clinical deterioration, demonstrating the urgent need for new therapeutical interventions.^10^

The oceans cover more than 70% of the Earth’s surface and can offer an enormous, previously unexplored chemodiversity. Marine ecosystems include habitats with physical and chemical characteristics that are considerably different from those on land.^11^ To adapt to extreme habitat conditions, marine organisms can develop unusual metabolic pathways and provide complex chemical structures of biotechnological interest.^12^ Microbial secondary metabolites play a highly significant role in the drug discovery and development process.^13^ Genetically, the chemical structures of microbial secondary metabolites are encoded by groups of microbial genes (Biosynthetic Cluster’s Genes).^14^ With the advancement of genomic technologies and the sequencing of the first microbial genomes of *Streptomyces* and *Aspergillus* published in the 2000s, it was shown that these microorganisms have in their genetic machinery the ability to produce a significantly higher number of compounds, with variations from one microorganism to another strain.^15,16^ These groups of genes are generally considered cryptic and are usually not expressed under laboratory conditions.^17^ Thus, triggering the expression of these cryptic genomic groups can result in an increased chemical diversity, allowing the discovery of new molecules of biological interest.^18^ Various methods have been developed for activating groups of cryptic genes that are poorly expressed. There are approaches that aim to modify the entire metabolome of the strain by generating pleiotropic effects to randomly activate any metabolic pathway. These approaches are technically simple and thus suitable for high-throughput scale-up.^17^ Several parameters must be considered when conducting experiments related to the production of microbial metabolites, such as the composition of the culture medium, pH, temperature, and oxygen, which can affect the metabolism of microorganisms and, consequently, the production of compounds.^19^ These parameters can be assessed using the OSMAC (one strain many compounds) approach, where a series of culture conditions are tested with the aim of generating different metabolites.^20^

Considering the therapeutic limitations of Chagas disease, there is an urgent need for the development of new drugs. Based on the high chemodiversity of metabolites produced by marine bacteria, these resources could be exploited to produce new pharmaceutical prototypes against neglected diseases. Therefore, the main objective of this study is to evaluate the *in vitro* anti-*Trypanosoma cruzi* potential of metabolites from the bacterium *M. indicus*, using the OSMAC approach.

## 2 Results

### 2.1 Isolation and Identification of the Bacterium from *Dichotomaria huismanii* Seaweed

A bacterium was isolated from the seaweed *Dichotomaria huismanii* and submitted to MALDI-ToF/MS analysis. The fingerprint of proteins was compared to the internal library of the equipment and resulted in the identification of a *Metabacillus indicus* strain, with scores >2. *Escherichia coli* was used as a standard and resulted in scores above 2.

### 2.2 Determination of the 50% Effective Concentration (EC_50_) of *Metabacillus indicus* Metabolites

The microbial extract of *Metabacillus indicus* (Mi) was evaluated against *T. cruzi* trypomastigotes under different OSMAC approaches. For comparison, the bacterium was grown in Marine Broth medium (static, 25 °C) and used as a control (Mi^a^), resulting in an EC_50_ value of 28.6 μg/mL.

#### 2.2.1 Physical Alteration of Cultivation

The cultivation of *M. indicus* at different physical conditions resulted in extracts with potent anti-*T. cruzi* activity in all extracts, with EC_50_ values ranging from 3.4 to 26.4 μg/mL (Table 1). The cultivation of *M. indicus* in liquid versus solid medium, resulted in extracts with EC_50_ values ranging from 3.4 to 28.6 μg/mL. The extract of metabolites produced by *M. indicus* cultivated in Marine Agar medium, resulted in an EC_50_ of 3.4 μg/mL (*p* < 0.05) at 25 °C (Mi^b^), but when cultivated at the same conditions at 40 °C, the EC_50_ resulted reduced to 11.8 μg/mL (Mi^d^) (*p* < 0.05). The bacterial metabolites obtained in Marine Broth (static) at 40 °C, resulted in an EC_50_ value of 14.9 μg/mL (Mi^c^). The shaking at 120 rpm at 25 °C in liquid medium resulted in an extract with an EC_50_ value of 8.4 μg/mL (Mi^e^)(*p* < 0.05). When *M. indicus* was cultivated at 4 °C (Mi^f^ and Mi^g^), no growth could be detected. The gamma irradiated *M. indicus*, cultivated in the liquid medium (Marine Broth), resulted in extracts with antitrypanosomal activity in a range between 11.7 and 26.4 μg/mL. The dose at 0.5 kGy resulted in the EC_50_ value of 11.7 μg/mL. Benznidazole was used as standard drug and resulted in an EC_50_ value of 5.0 ± 0.7 μg/mL. The physical alterations in *M. indicus* cultivation resulted in different yields (total mass of metabolites). The cultivation in solid medium (agar) at 25 °C resulted in 2 mg of crude extract, and at 40 °C, it resulted in 11 mg. In the liquid medium (Marine Broth), the cultivation at 25 °C under shaking at 120 rpm, yielded 1.7 mg, but when irradiated at 0.5, 1.5, and 3 kGy, the yield was 0.6, 1.7, and 9.7 mg (Table 1).

**Table 1:** Physical Alterations for Growth Conditions of *M. indicus*^a^^b^

Extract	Cultivation condition	Amount of extract (mg)	EC_50_ ± SD (μg/mL)
Mi^b^	Marine Agar at 25 °C	2.0	3.4 ± 0.1*
Mi^c^	Marine Broth static at 40 °C	3.8	14.9 ± 0.1*
Mi^d^	Marine Agar at 40 °C	11.0	11.8 ± 5.0*
Mi^e^	Marine Broth—shaking 120 rpm at 25 °C	1.7	8.4 ± 0.6*
Mi^f^	Marine Agar at 4 °C	0	ND^1^
Mi^g^	Marine Broth static at 4 °C	0	ND^1^
Mi^h^	Irradiation at 0.5 kGy in Marine Broth static at 25 °C	0.6	11.7 ± 2.2*
Mi^i^	Irradiation at 1.5 kGy in Marine Broth static at 25 °C	1.7	15.5 ± 3.8
Mi^j^	Irradiation at 3 kGy in Marine Broth static at 25 °C	9.7	26.4 ± 4.5

a EC_50_; 50% effective concentration (μg/mL), SD; standard deviation, *p < 0.05; ND^1^- not determined, *M. indicus* produced no growth at this temperature.

b Evaluation of anti-*Trypanosoma cruzi* activity of metabolites against trypomastigotes with respective yields.

#### 2.2.2 Nutritional Alteration of Cultivation

Nutritional alterations of *M. indicus* medium resulted in extracts with potent anti-*Trypanosoma cruzi* activity, with EC_50_ values between 11.0 and 18.4 μg/mL (Table 2). The extract obtained from the growth in Mueller Hinton Broth medium showed an EC_50_ value of 11 μg/mL, while in Trypsin Soy Agar, an EC_50_ value of 12.5 μg/mL. When grown in R2A medium, the bacterial extract resulted in an EC_50_ value of 18.4 μg/mL. *Metabacillus indicus* showed no growth when cultivated in A1 medium (broth and agar) as well as in Malt Agar. The mass yield of metabolites from the *M. indicus* extract was 9.1 mg in Mueller Hinton, 2.7 mg in TSA, and 0.6 mg in R2A.

**Table 2:** Nutritional Alterations of the Growth Conditions of *M. indicus*^a^^b^

Extract	Cultivation condition	Amount of extract (mg)	EC_50_± SD (μg/mL)
Mi^k^	Trypsin Soy Agar (TSA)	2.7	12.5 ± 2.5*
Mi^l^	R2A	0.8	18.4 ± 1.5
Mi^m^	Mueller Hinton Broth	9.1	11.0 ± 1.1*

a EC_50_; 50% effective concentration (μg/mL), SD; standard deviation, *p < 0.05.

b Evaluation of anti-*Trypanosoma cruzi* activity of metabolites against trypomastigotes with respective yields.

#### 2.2.3 Biological Alterations of Cultivation

Biological variations of growth induced modifications to the potency of the extracts against *T. cruzi*, with EC_50_ values ranging from 11 to 28 μg/mL (Table 3). These alterations were carried out using cocultivation with other marine and terrestrial bacteria, as well as the addition of parasitic antigens associated with the medium. When *M. indicus* was cocultivated with a multidrug-resistant *Acinetobacter baumannii*, the extract (containing *M. indicus* only) demonstrated an EC_50_ of 15 μg/mL (Mi^q^). Co-cultivation with multidrug-resistant *Sg*, it showed an EC_50_ of 19 μg/mL (Mi^p^). Co-cultivation in the presence of the marine strains such as *Planococcus maritimus* and *Halomonas aquamarina* afforded EC_50_ values of 17 (Mi^n^) and 16 μg/mL (Mi^o^), respectively. When co-cultured in liquid medium with another marine strain, *Vibrio furnissii*, the extract of the mixture *M. indicus* + *V. furnissii*, resulted in metabolites with an EC_50_ of 28 μg/mL (Mi^r^). The extract of *V. furnissii* (grown in static Marine Broth at 25 °C) was prepared to verify the anti-*T. cruzi* activity. We obtained an EC_50_ value of 28 μg/mL, similar to that of the Mi^a^ extract cultivated under the same conditions. When cultivated in association with *T. cruzi* and *Leishmania infantum* antigens, the extract showed EC_50_ values of 11 μg/mL (Mi^t^) and 11.5 μg/mL (Mi^s^), respectively. Considering the biological alterations, the total yield of extracts ranged from 1.3 to 6.5 mg. The yield of metabolites in co-culture with *A. baumannii* was 1.3 mg and with *S. aureus*, 1.4 mg. The yields obtained in the presence of *T. cruzi* and *L. infantum* antigens were 1.4 and 1.3 mg, respectively. With the marine strains *P. maritimus* and *H. aquamarina*, *M. indicus* resulted in yields of 1.9 and 2.2, respectively. Finally, the yield obtained from liquid cultivation with *M. indicus* + *V. furnissii* was 6.5 mg.

**Table 3:** Biological Alterations of the Growth Conditions of *M. indicus*^a^^b^

Extract	Cultivation condition	Amount of extract (mg)	EC_50_± SD (μg/mL)
Mi^n^	Marine Agar + *Planococcus maritimus*	1.9	17.1 ± 6.1
Mi^o^	Marine Agar + *Halomonas aquamarina*	2.2	16.0 ± 3.2
Mi^p^	Marine Agar + *Staphylococcus aureaus*	1.4	19.0 ± 2.1
Mi^q^	Marine Agar + *Acinetobacter baumannii*	1.3	15.0 ± 0.9*
Mi^r^	Marine Broth + *Vibrio furnissii*	6.5	28.0 ± 2.0
Mi^s^	Marine Agar + antigen of *L. infantum*	1.3	11.5 ± 3.0*
Mi^t^	Marine Agar + antigen of *T. cruzi*	1.4	11.0 ± 6.4*

a EC_50_; 50% effective concentration (μg/mL), SD; standard deviation, *p < 0.05.

b Evaluation of anti-*Trypanosoma cruzi* activity of metabolites against trypomastigotes with respective yields.

#### 2.2.4 Chemical Changes

For this section, the extract of *M. indicus* cultivated in Marine Broth with orbital agitation at 120 rpm (Mi^e^) was used as a control for comparison purposes. This extract resulted in an EC_50_ value of 8.4 μg/mL in a yield of 1.7 mg. Chemical elicitors such as EtOH and DMSO were incubated with *M. indicus* under orbital agitation at 120 rpm and resulted in extracts with EC_50_ values of 17 and 22 μg/mL, respectively. In the presence of DMSO, a higher amount of extract was observed with a yield of 27 mg, but in the presence of EtOH, the yield was 3.8 mg (Table 4).

**Table 4:** Chemical Alterations of the Growth Conditions of *M. indicus*^a^^b^

Extract	Cultivation condition	Amount of extract (mg)	EC_50_± SD (μg/mL)
Mi^u^	Marine Broth +3% ethanol + shaking 120 rpm	3.8	17.0 ± 0.6
Mi^v^	Marine Broth +3% DMSO + shaking 120 rpm	27.0	22.1 ± 2.0

a Evaluation of anti-*Trypanosoma cruzi* activity of metabolites against trypomastigotes with respective yields.

b EC_50_; 50% effective concentration (μg/mL); SD: standard deviation.

### 2.3 Chemical Dereplication of Metabolites of *M. indicus*

The chemical profile of *M. indicus* was initially analyzed by ^1^H NMR which showed signals corresponding to different classes of metabolites such as nucleosides, peptides, steroids, and fatty acids. Sequentially, this material was analyzed by UHPLC-ESI-HR-MS/MS in positive ion mode. The high-resolution mass spectra were organized and processed using the GNPS platform with network visualization managed through Cytoscape software. This process led to the annotation of different mass spectra, related to the compounds previously detected by NMR, as indicated in Table 5.

**Table 5:**
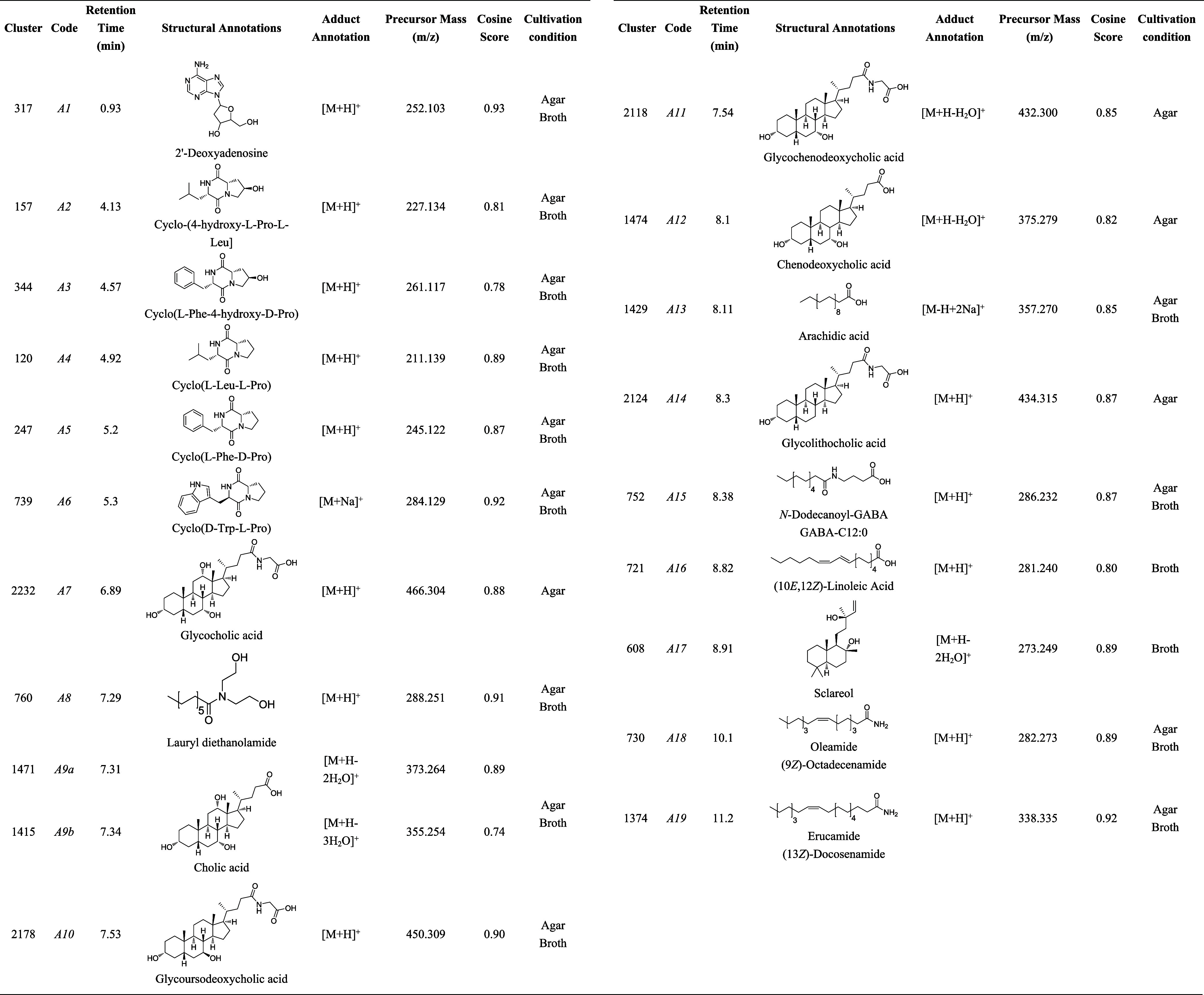
Annotations Obtained from GNPS Dereplication Workflow of UHPLC-ESI-HR-MS/MS Analysis of Secondary Metabolites of *M. indicus* Strain from the Incubation in Marine Agar (Mi^b^) and Marine Broth Static (Mi^a^)

Several metabolites were identified, including 2’-deoxyadenosine (A1), diketopiperazine derivatives (A2-A6), and fatty acid derivatives (A8, A13, A15, A16, A18, A19). The primary structural annotations belonged to the terpenoid class of steroids, specifically cholic acid derivatives, such as glycocholic acid (A7), cholic acid (A9), glycoursodeoxycholic acid (A10), glycochenodeoxycholic acid (A11), chenodeoxycholic acid (A12), and glycolithocholic acid (A14), along with a single labdane diterpenoid, sclareol. Notably, the *M. indicus* strain was capable of producing glycocholic, glycochenodeoxycholic, chenodeoxycholic, and glycolithocholic acids exclusively during Marine Agar incubation, whereas linoleic acid and sclareol were detected only during Marine Broth static growth. These findings suggested that different culture media and conditions induced distinct metabolomic profiles in *M. indicus*.

**Figure 1:**
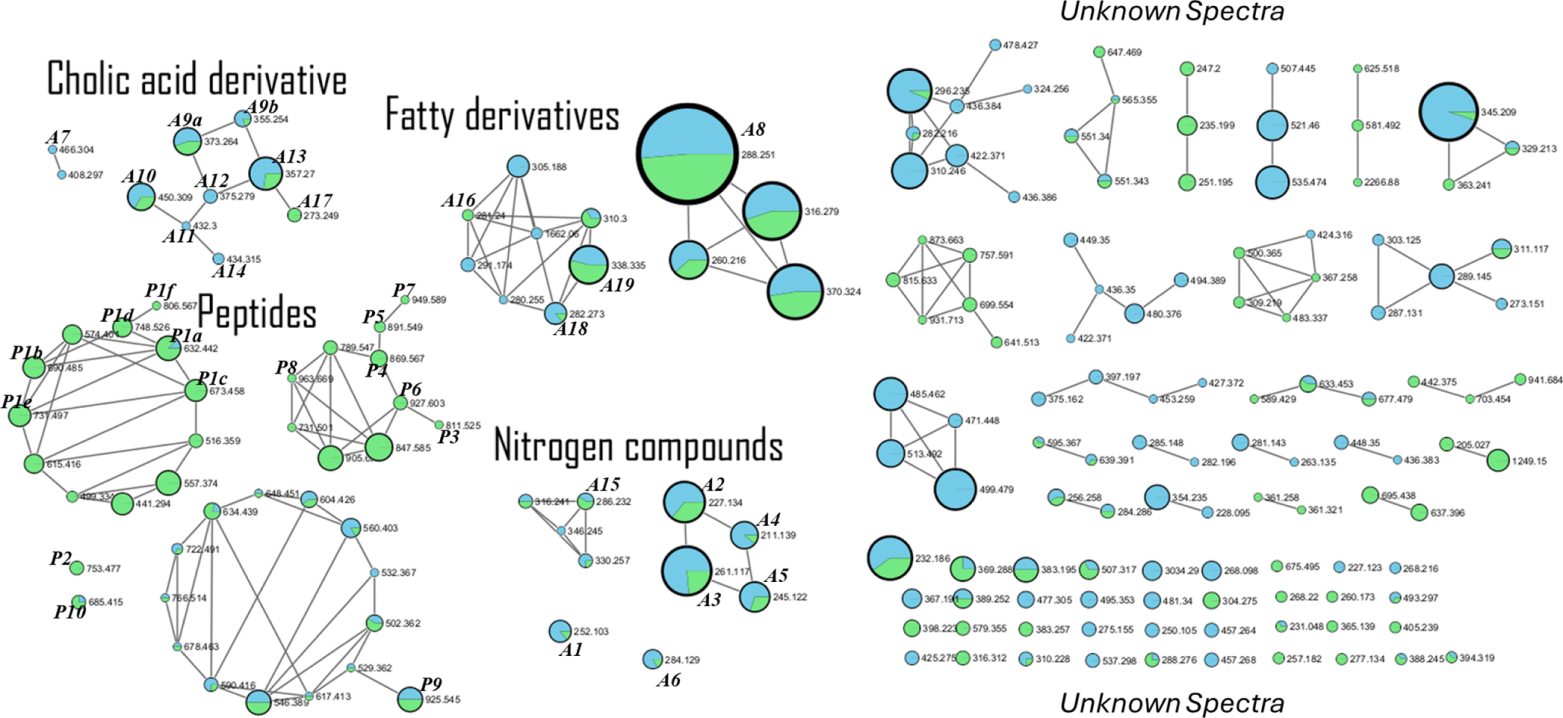
Classical molecular networking from UHPLC-ESI-HR-MS/MS acquisition. The connection between nodes represents a similarity between fragments (cosine above 0.5). The colors inside node represent the spectra percentage detected in Marine Agar (blue) and Marine Broth (green) incubation. The node size varies from 35 spectra clustered (biggest node, A8 - lauryl diethanolamide) to 2 spectra clustered (smaller nodes).

Peptides were annotated using the *in-silico* tool Dereplicator, and analogues were identified through VarQuest. The annotated peptides with significant matches included debromo-35,36-dihydro-veraguamide A (P1a to P1f, various adducts in the same molecular family; Figure 1), galantin 1 (P5), 7,7,8,8-tetrahydro wewakpeptin A (P6), and dolastatin 12 (P7) (structures shown in Table 6). Among the spectral families with peptide annotations, six additional peptide MS^2^ spectra were annotated as unreliable, according to the GNPS Dereplicator workflow, but coherent to the network connections (Table 6). The connections between two or more nodes, representing similar MS^2^ spectra between molecular ions, provided significant information about structural similarities, indicating the presence of potential peptide structures, supported by peptide annotations from Dereplicator and VarQuest.

**Table 6:**
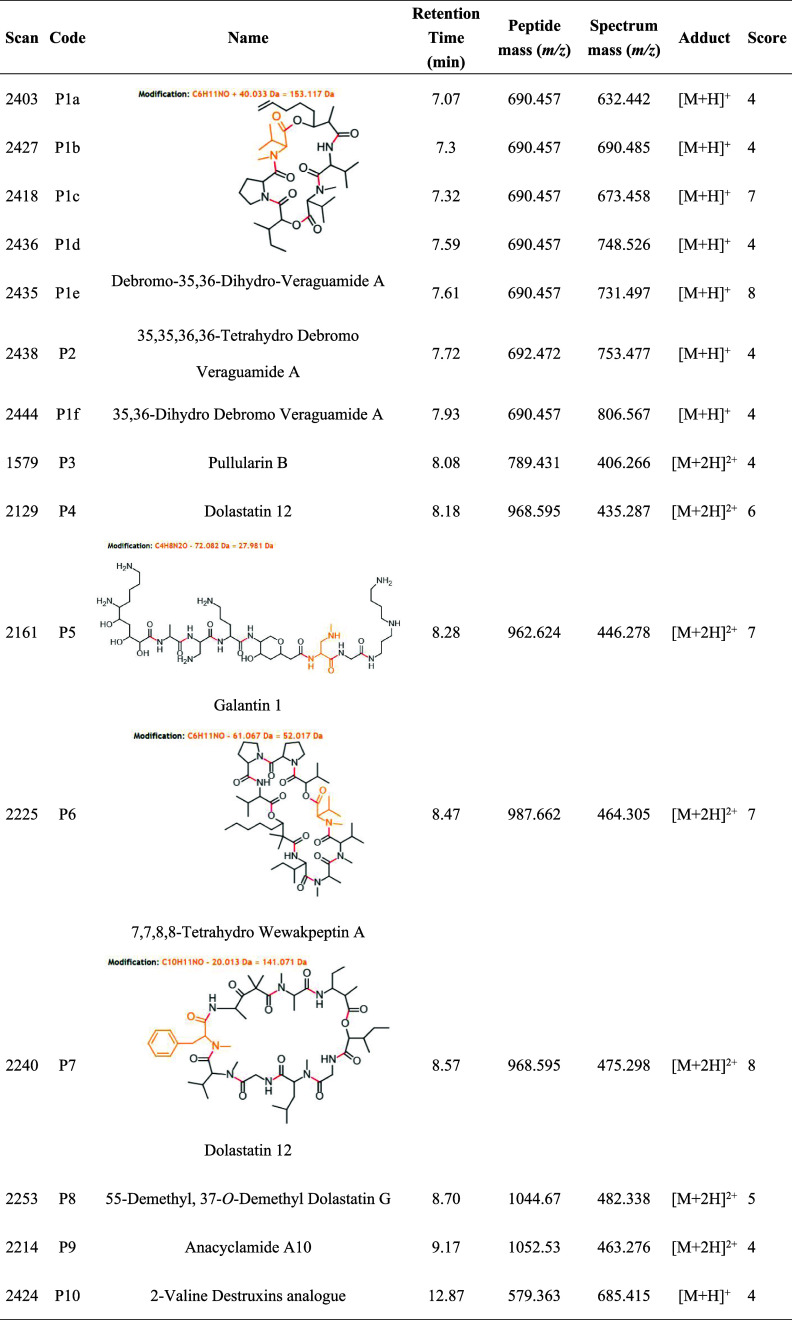
Peptide Annotations Obtained from the Dereplicator VarQuest *In Silico* Tool from the GNPS Platform, Based on the *Metabacillus indicus* Molecular Networking Workflow^a^

a The most reliable annotations contain structural annotations, including the modification suggested by dereplicator.

## 3 Discussion

Currently affecting 21 countries, Chagas disease (CD) is no longer a problem limited to the American continent. As a potentially fatal disease, CD needs chemotherapeutical interventions, but in Brazil, only one single drug is available. Due to the severe adverse effects and extremely limited efficacy of benznidazole, there is an urgent need for new drug candidates. The marine environment is considered to be a rich source of numerous compounds with antibacterial, antifungal, antiviral, and antiparasitic activities.^21^ In a short period between 1977 and 1987, more than 2500 new small molecules were discovered from marine organisms.^22^ In nature, microorganisms are pressured by physical and chemical environmental challenges but are also involved in a dynamic network of intra- and interspecies interactions, leading to the production of a myriad of secondary metabolites. In our study, we observed for the first time the anti-*Trypanosoma cruzi* activity of metabolites from the marine bacterium *Metabacillus indicus*. *Metabacillus indicus* (former *Bacillus indicus;Bacillus cibi)*, belongs to the genus *Bacillus*, a promising group of Gram-positive bacteria widely found in terrestrial and marine environments.^23^ The antimicrobial potential of *M. indicus* has been underexplored, with a single report demonstrating the activity of the metabolites against bacterial biofilms.^24^ An antioxidant flavonoid named xanthorhamnin was isolated from *M. indicus* and demonstrated antitumor activity, as well as UV radiation resistance.^25^ Due to its promising pharmaceutical potential, this strain was selected by our group to be cultivated under different conditions (OSMAC approach), aiming for the first time for the production of potential compounds against *Trypanosoma cruzi*.

According to Martín and coworkers,^26^ the selection of culture media can directly influence the expression of metabolites, since different compositions provide specific conditions for the growth and metabolism of microorganisms. In our work, the extract of *M. indicus* metabolites, produced in Marine Broth medium (static growth at 25 °C), was selected as an initial condition. When compared to other available bacterial media, Marine Broth is a complex medium, providing the bacteria, with sources of nitrogen, boron, chlorine, silicate, bromine, calcium, magnesium, strontium, and peptone, as well as an undefined yeast extract. The EC_50_ value obtained in this condition was used for comparisons to all results obtained with modified conditions of growth (except to chemical alterations).

As a starting phase of the OSMAC approach, nutritional variations were offered to *M. indicus*. When cultivated in TSA medium, the marine bacteria produced metabolites with 2.2-fold (*p* < 0.05) higher antitrypanosomal activity. When cultivated in Mueller–Hinton broth medium, the metabolites showed 2.6-fold (*p* < 0.05) higher antiparasitic activity, but no significant improvement in activity could be found when *M. indicus* was cultivated in R2A medium. Analyzing the compositions of these media, variations can be observed in the sources of nitrogen and carbon as well as different quantities of nutrients. In R2A and Marine Broth (or agar), the primary nitrogen source is peptone, with higher amounts in Marine medium. The R2A contains hydrolyzed casein, which is absent in the Marine medium. In TSA, the main nitrogen source is tryptone, which is a product of the pancreatic digestion of casein. Regarding the main carbon sources, Marine has yeast extract and R2A, a combination of yeast extract, dextrose, and starch but in different proportions compared to Marine medium. In TSA, soybean is considered to be the carbon source. In Mueller–Hinton medium, the main nitrogen sources are casein hydrolyzate and beef extract, while the carbon source is starch. These results suggest that the differences in the composition and amount of nutrients in mediums can directly impact the production of antitrypanosomal metabolites by the marine *M. indicus*. These results corroborate other studies,^17^ as the *Vibrio* strain, where nutritional alterations demonstrated strong influence in the antimicrobial potency of the bacterial metabolites. According to this study, five BGCs in the genome of a *Vibrio* strain were differentially expressed when grown with chitin rather than glucose, with an increased production of the antibiotic andrimide in cultures.^27^

No growth was observed when *M. indicus* was cultivated in Malt agar and A1 mediums. In the A1 medium, tryptone and lactose are the sources of nitrogen and carbon. Additionally, this medium contains surfactant Triton X-100, which may have hindered the growth of *Metabacillus indicus*. The malt extract provides nutrients, such as carbohydrates, amino acids, and vitamins, but these may have been insufficient for the growth of *Metabacillus indicus*. Similarly, the acidic pH of Malt agar (pH 5) may have affected bacterial growth, given that the pH of the standard medium (Marine) is 7.

In the OSMAC approach, the cocultivation of bacteria has been reported as a relevant factor for the production of new bioactive metabolites.^17^ Sung and coworkers^28^ cocultured marine *Streptomyces* sp. with *S. aureaus*. Their results showed an increase in the production of three compounds with antimicrobial activity, granaticin, granatomycin D, and dihydrogranaticin B, suggesting that competitive interactions can alter the production of metabolites. Our second OSMAC approach involved the cocultivation of *M. indicus* with a living multidrug-resistant bacterium *Acinetobacter baumannii*, which resulted in metabolites with 1.9-fold more antitrypanosomal potency. The co-cultivation with other marine strains *Planococcus maritimus*, *Halomonas aquamarina*, and *Vibrio furnissii*, as well as with the multiresistant bacteria *Staphylococcus aureus*, showed no improvements in the antitrypanosomal potential, with similar EC_50_ values to the standard condition. Co-cultivation with parasites (lysates of *Leishmania* and *T. cruzi*) as a source of antigens, resulted in a 2.5-fold higher potency, suggesting a differentiated production of metabolites by *M. indicus* in the presence of parasitic cell contents. This approach could influence the initiation processes of bacterial cell machinery and also the expression of cryptic genes, to increase competitiveness and fitness, which can often lead to the enhanced production or even the development of new bioactive secondary metabolites.^29^

Chemical elicitors are compounds that can alter the production of secondary metabolites, causing changes in the translation of proteins in bacteria, and possibly inducing a response to stress, which can unblock other metabolic pathways.^30^ As the third stage of our OSMAC study, *M. indicus* was cultivated under the pressure of the chemical elicitors DMSO and ethanol. Considering the standard conditions for this assay (cultivation in Marine Broth with shaking), the obtained data showed that both elicitors (ethanol and DMSO) resulted in significant losses of the antitrypanosomal potency of *M. indicus* metabolites. These data suggest that both chemical elicitors may have altered the expression of the antiparasitic metabolite(s) present in the *M. indicus* extract, affecting the potency against the trypomastigotes. In contrast, the cultivation of the marine fungus *Pestalotiopsis* sp. with ethanol, led to the synthesis of benzophenone, a new antimicrobial compound.^31^ Chemical elicitation studies with bacterial strains under the pressure of DMSO showed significant qualitative and quantitative changes in the production of antimicrobial metabolites.^32,33^

Differential metabolite production has been described for bacteria when they are cultivated under diverse physical conditions. As the fourth stage of our OSMAC approach, we studied temperature variation, a condition that can affect both cell proliferation rates and the secondary metabolism of bacteria.^34^ When *M. indicus* was shifted from the standard growth at 25–40 °C, using static Marine Broth and Marine Agar, a significant (*p* < 0.05) increase (2-fold) in the antiparasitic activity was observed against the trypomastigotes. In the literature, studies have been shown that certain metabolites are only produced in certain temperature ranges.^35,36^ In our study, the increased temperature may have accelerated the bacterial metabolism or gene expression of the anti-*Trypanosoma cruzi* metabolite(s) of *M. indicus*.

Changes from static to shaking cultivation are factors that definitely contribute to the production of secondary metabolites, providing aeration and higher contact with nutrients in the culture medium.^17^ In our study, *M. indicus*, when cultivated in Marine Broth medium under orbital agitation, significantly (*p* < 0.05) enhanced 3.4-fold the antitrypanosomal activity. Studies carried out with *Pseudomonas putida* showed that the culture conditions (static and under agitation) altered the genotoxic potential of metabolites produced by the bacterium, which were potentiated in the static growth.^37^ Studies with the deep-sea fungi *Penicillium* sp. demonstrated the production of five new sorbicillinoids under agitation conditions.^38^

γ Radiation can be used as a biotechnological tool to induce random mutagenesis in microbial strains to increase the production of certain products. In our study, *M. indicus* irradiated with cobalt-60 at a nonlethal dose of 0.5 kGy, produced anti-*T. cruzi* metabolites with 2-fold higher potency than the standard strain. γ Radiation can directly affect the cells, interacting with DNA, proteins, membranes, and organelles but also through an indirect effect, the water radiolysis. This process breaks down the water to produce electrons, H and OH radicals, H_3_O^+^ and different molecules (H_2_ and H_2_O_2_), affecting cells by different mechanisms.^39^ Khaliq and coworkers demonstrated that a gamma-irradiated *Streptomyces* spp. can produce 5-fold higher amounts (2,500 mg/L) of the antimicrobial compound tylosin, when compared to the wild strain (550 mg/L).^40^ Similarly, the antibiotic oxytetracycline, produced by a *Streptomyces* spp., has been shown to be increased by 19-fold when irradiated with γ rays.^41^

English and colleagues showed that antimicrobial compounds produced by *Streptomyces* spp. in solid medium were absent in the liquid medium.^42^ As a final OSMAC alteration, *M. indicus* was cultivated in marine agar (solid medium) and demonstrated the most promising condition. It produced an extract with approximately 8-fold higher antitrypanosomal potency than those obtained under the standard conditions of cultivation (Marine Broth). The data analysis suggests that some conditions could have contributed to the potentiation of the extracts, including: i) the presence of agar in the culture medium; ii) the higher aeration in the solid growth; iii) the cultivation vessel allowing the formation of biofilm. Agar is a heterogeneous mixture of two polysaccharides, agarose and agaropectin, which contribute to bacterial metabolism during growth.^43^ This widely used solidifying agent could also have served as an additional nutrient for *M. indicus*, providing excellent nutritional conditions in the culture media for the production of different metabolites.

*Bacillus* spp. can form biofilms, a mode of collective living that confers emergent properties to the inhabitants of these communities.^44^ Biofilm formation is a defense mechanism for bacteria, inducing the production of different compounds for self-protection.^45^ The cultivation of *M. indicus* as a “biofilm” may have contributed to the production of antitrypanosomal compounds.

The oxygen concentration on bacterial growth has a known impact in metabolites production. Cultivation vessels can also affect aeration and stimulate the growth of bacteria, increasing the production of metabolites with antimicrobial properties.^17^ In our study, the cultivation of *M. indicus* (Marine Agar) in a biological oxygen demand incubator (forced air circulation) in 150 × 15 mm Petri plates may have contributed to a substantial aeration when compared to the growth in the liquid medium, using a static condition. Fan and coworkers demonstrated that extracts produced by 10 different strains of fungi in liquid and solid culture, shared only 24% of chemical compounds. Furthermore, 30% of these metabolites were only produced under a single culture regimen, as observed by NMR and HRMS-MS associated with GNPS molecular network analyses.^46^ A study carried out with a marine strain *Streptomyces* spp. demonstrated the effects of hypoxia on the cultivation of the bacteria associated to the production of secondary metabolites; the bacterium produced the intermediate metabolite 8-amino-flaviolin instead of the antibiotic napiradiomycin.^47^

It was noteworthy that our *in vitro* studies confirmed differences of the biological activity of metabolites produced by *M. indicus* under different cultivation methodologies. To confirm the hypothesis that the OSMAC approach rendered differential production of metabolites, we analyzed the metabolites under two bacterial growth conditions: solid (Marine Agar) and liquid (Marine Broth) under static. The GNPS analysis showed several substances without annotation in the database, suggesting possible new compounds in the *M. indicus* extract. In general, the results suggested the presence of different compounds, such as nucleosides, peptides, steroids, and fatty acid derivatives.

In a search for the biological activities of compounds annotated in the *M. indicus* extracts, the literature shows promising antiparasitic, antimicrobial, antifungal, and anticancer properties. In our analysis, a single nucleoside (A1) was annotated, named 2’-deoxyadenosine. Anticancer activity in lymphoblastic leukemia cells have been reported for 2-chloro-2’-fluoro-2’-deoxyarabinosyladenine, a 2’-deoxyadenosine analogue.^48^

Our data demonstrated that compounds such as glycocholic (A7), glycochenodeoxycholic (A11), chenodeoxycholic (A12), and glycolithocholic (A14) acids were exclusively annotated in the solid cultivation, whereas linoleic acid and sclareol were detected only during the growth in Marine Broth (static). The metabolites produced under the solid growth demonstrated the highest antitrypanosomal potency when compared to those obtained in the liquid (static) growth. Glycocholic acid (A7) was previously isolated from the aquatic bacteria *Bacillus amyloliquefaciens* and demonstrated antimicrobial activity against *P. aeruginosa* (250 μM) and *B. cereus* (15.6 μM),^49^ Indeed, *Trypanosoma cruzi* parasites have been demonstrated susceptibility to steroids as well as to fatty acids. A chemoinformatic analysis conducted between 2019 and 2024 on a data set of natural products with antiprotozoal activity, showed steroids as promising compounds.^50^ Telocinobufagin and hellebrigenin, two steroids isolated from a Brazilian toad, presented activity against *T. cruzi* trypomastigotes and *Leishmania infantum*.^51^ The steroids glycoursodeoxycholic acid (A10) and glycochenodeoxycholic (A11) acid have been shown anticancer activity against hepatoma carcinoma HepG2 cells^52^ and antimicrobial activities against *Staphylococcus aureus*, *Pseudomonas aeruginosa*, *Staphylococcus epidermidis*, and *Escherichia coli*, respectively.^53^ The biosynthesis of cholic acid derivatives by a marine bacterium was previously reported;^54^ this compound (A9) was also annotated in our study. Antiparasitic activity of cholic acid derivatives has been reported against *Trypanosoma brucei* and *Plasmodium*.^55^ Synthetic derivatives of chenodeoxycholic acid (A12) have also been shown promising activity against *T. cruzi*, with EC_50_ values in a range between 8.6 and 22.8 μM.^56^ The single labdane diterpenoid sclareol, annotated as compound A17, has been shown antimicrobial activity against *Staphylococcus aureaus*, with MIC values ranging from 0.09 to 0.74 mg/mL.^57^

Fatty-acids have shown promising antiparasitic activities in the literature, compounds that were also annotated in *M. indicus* extract. Linoleic acid (A16), demonstrated activity against the nematodes *Brugia malayi*, *Brugia timori*, and *Wuchereria bancrofti*, with an EC_50_ value of approximately 4 μg/mL.^58^ The long-chain fatty acid arachidic acid (A13) has been shown anti-*Leishmania donovani* activity, with a potent EC_50_ value of 5 μg/mL.^59^ Other fatty acids as oleamide (A18), a fatty amide derived from oleic acid, has been shown antimicrobial activity against *Bacillus subtilis* and *Escherichia coli*;^60^ erucamide (A19) demonstrated promising antiparasitic activity against *Plasmodium falciparum* (EC_50_ value of 32 μM).^61^ Other marine bacteria, *Mesoflavibacter zeaxanthinifaciens*, has been shown iso-type fatty acids with potent activity against the extracellular and intracellular forms of *T. cruzi*.^62^

Diketopiperazines are characterized by a six-membered bis-lactam ring, whose carbon and nitrogen atoms can be modified to produce various derivatives. Diketopiperazines can present a wide range of structural diversity and biological properties, including anti-*T. cruzi* activity.^63^ In our study, five diketopiperazines were identified (A2–A6). In the literature, the diketopiperazine (A4) was previously isolated from a marine bacterium from the *Bacillus* genus but demonstrated no activity against the amastigote forms of *Trypanosoma cruzi*.^64^ Compound A2 (cyclo-(4-hydroxy-l-Pro-l-Leu) isolated from the deep-sea-derived actinomycete *Micrococcus* sp. showed anticancer activity in RAW264 cells.^65^ Additionally, compound A3 (cyclo(l-Phe-4-hydroxy-d-Pro)) has also been shown anticancer activity against human glioma cells U87-MG and U251, with EC_50_ values of 5 and 18 μM, respectively.^66^

A higher diversity of peptides was observed in the *M. indicus* extract when the bacteria were grown in a liquid medium. In recent years, a variety of lipopeptides, nonribosomal peptides, and glycopeptides have been isolated from marine *Bacillus* specimens, showing diverse biological activities.^67−69^ Although marine peptides are poorly explored, these compounds have intrinsic activity and the ability to inhibit microorganisms, and could be valuable sources of new pharmaceutical prototypes.^70^ Marine peptides are structurally diverse and shows a wide range of therapeutic actions with high target-specificity.^71^ In the literature, peptides have been shown activity against different strains of *T. cruzi*, exerting specific effects through different mechanisms, such as disruption of the plasma membrane, alteration of calcium homeostasis, inhibition of some metabolic pathways, disruption of organelles and activation of various cell death pathways.^72^ To the best of our knowledge, no anti-*T. cruzi* activity has been reported for the peptides annotated in our study. However, in the literature, peptides isolated from cyanobacteria have been shown anticancer properties. Veraguamide A (P2) has demonstrated potent cytotoxicity for the human lung cancer cell line H-460,^73^ while dolastatin 12 (P7), an anticancer activity against adenocarcinoma and mammary adenocarcinoma.^74^ Wewakpeptin A (P6) has also been shown activity against human lung tumor cell lines NCI-H460 and mouse neuroblastoma neuro-2a.^75^

Despite the small number of metabolites annotated in *M. indicus* that present anti-*Trypanosoma cruzi* activity, the literature reports a wide range of biological activities, including antiparasitic, antibacterial, antifungal, and anticancer effects. These data could provide important information on anti-*T. cruzi* compounds present in *M. indicus*, supporting the future isolation of the active compounds.

## 4 Materials and Methods

### 4.1 Parasites

Trypomastigotes of *T. cruzi* (strain Y) were maintained in Rhesus monkey kidney cells (LLC-MK2 - ATCC CCL 7), using RPMI-1640 medium supplemented with 3% fetal bovine serum (FBS), in a 5% CO_2_ incubator at 37 °C.^76^

### 4.2 Seaweed Collection and Isolation and Identification of the Marine Bacteria

The seaweed was collected in 2023 through scuba diving in the São Sebastião region (coordinates: S 23°49′40.7″W045°24′44.7″) with the assistance of Prof. Alvaro E. Migotto from CEBIMar USP (SISBIO Biodiversity Authorization and Information System 10186-2). The material was collected at a depth of 2 m, in minimal quantities to avoid any environmental impact, and identified at the Biosciences Institute by Prof. Mariana Cabral and Prof. Valeria Cassano (University of Sao Paulo, Brazil). The seaweed was collected and immediately processed under sterile conditions. The surface decontamination was performed with 70% EtOH followed by washing in sterile seawater. Using a sterile scalpel, a fragment of approximately 1 cm^2^ was excised, macerated in sterile seawater (1 mL), and inoculated onto 90 × 15 mm Petri dishes containing nutrient-rich medium (Marine Agar DIFCO) and a low-nutrient medium (agar medium in seawater).^77^

The endophytic bacterium was isolated from the seaweed and identified by MALDI-ToF/MS (matrix associated laser desorption-ionization time of flight). Briefly, the bacterial colonies were suspended in 300 μL of ultrapure water (Milli-Q), with 900 μL of EtOH. After centrifugation, 50 μL of 70% (v/v) formic acid and 50 μL of acetonitrile (100%) were added, and 1 μL was seeded into a 96-well plate for MALDI-ToF/MS and air-dried. The matrix α-cyano-4-hydroxy-cinnamic acid (HCCA) (Bruker-Daltonics) was used for mass spectrometry analysis. The analyses were performed on a Microflex mass spectrometer (Bruker-Daltonics) with a nitrogen laser (337 nm) operating in linear mode with delayed extraction (260 ns) at a 20 kV accelerating voltage. Each spectrum was automatically collected in positive-ion mode on average of 500 laser shots (50 laser shots at 10 different point positions). A mass range between 2000 and 20,000 *m*/*z* was selected to collect the signals with the Auto Xecute tool of the flexcontrol acquisition software (Version 2.4; Bruker-Daltonics). Score values above 2 indicate species-level identification of bacteria.^78^

### 4.3 Bacterial Cultivation and Extraction of Metabolites: Growth in Liquid and Solid Media

Cultivation was carried out in both liquid and solid media. In liquid media, the bacteria were grown statically or under agitation. A preinoculum was prepared and grown for 24 h in a BOD incubator at 25 °C. The secondary culture was inoculated at 1.5 × 10^8^/UFC. The cultures were kept in a BOD incubator at 25 °C for 10 days. The samples were centrifuged at 4000 g for 20 min, and the bacterial precipitate was washed in sterile seawater to remove possible metabolites from the culture medium. The samples were centrifuged again, and the supernatant was discarded. In solid media cultures, a 24 h preculture was carried out in 90 × 15 mm Petri dishes. The bacterium was inoculated in 150 × 15 mm Petri dishes, and the cultures were kept in a BOD incubator at 25 °C for 10 days. The bacterial mass was scraped off individually with a sterile cell scraper. After this stage, 200 mL of Milli-Q water was added to both cultures followed by 200 mL of EtOAc. The material was subjected to an ultrasound bath for 40 min at room temperature and transferred to a separating funnel to separate the aqueous phase from the organic phase. The EtOAc phase was filtered and the material was concentrated in vacuum at 40 °C to afford the organic crude extract which was stored at −20 °C.^79^

### 4.4 Evaluation of the Anti-*Trypanosoma cruzi* Activity

The 50% effective concentration% (EC_50_) of the extracts was evaluated on trypomastigote forms of *T. cruzi* (strain Y). The drug benznidazole was used as a standard. Untreated cells were used as negative controls (100% viability). To evaluate the antitrypomastigote activity, the microbial organic extracts were dissolved in methanol, diluted in RPMI-1640 medium at concentrations between 1.1 and 150 μg/mL. The trypomastigotes (1 × 10^6^ /well) were incubated for 24 h in a 5% CO_2_ incubator at 37 °C. The maximum concentration of solvent per well was 0.5% (v/v) to avoid cell toxicity. The viability of the trypomastigotes was assessed using the resazurin colorimetric method^80^ and monitored in a spectrophotometer (FilterMax F5 Multi-Mode) at λ 570 nm.

### 4.5 OSMAC Approach

The marine strain *Metabacillus indicus* was cultivated under different conditions, and the strains were subdivided into four groups.

#### 4.5.1 Changes of Physical Parameters

Temperature, using a BOD incubator at 27 °C, a refrigerator at 4 °C, and a bacteriological incubator at 40 °C, growth in liquid and solid media, static cultivation, and under orbital agitation at 120 rpm, as well as irradiation of the strain with ^60^Co γ rays. *Metabacillus indicus* was irradiated to induce random mutagenesis, and the experiment was carried out at the Nuclear and Energy Research Institute (IPEN/CNEM-SP) at 25 °C in an ice bath to avoid heating, using a ^60^Co-gamma ray source at a rate of 1 kGy/h. The doses used were 0.5, 1.5, and 3 kGy. The strain was irradiated in static Marine Broth with 1 day of preinoculum and then transferred to an incubator for an additional 9 days incubation. After irradiation, the strain was grown in Petri dishes in Marine Agar medium to assess the viability.

#### 4.5.2 Changes of Nutritional Parameters

Cultivation in media with different compositions aimed at the variation of nutrient sources and concentrations. The solid mediums were Marine (Difco), Trypsin Soy Agar (Kasvi), A1 (Difco), Malt Agar (Neogen), and R2A Agar (Difco), and liquid mediums were Marine (Difco), Mueller Hinton Broth (Sigma-Aldrich), and A1 (Difco).

#### 4.5.3 Changes of Biological Parameters

Co-cultivation with different bacterial strains in solid medium with *Planococcus maritimus*, *Halomonas aquamarina*, *Staphylococcus aureaus* (ATCC 3591), the multidrug-resistant bacteria *Acinetobacter baumannii* (261/16), and the marine bacteria *Vibrio furnissii* in liquid medium. *Vibrio furnissii* was cultivated in static Marine Broth at 25 °C and the organic extract was prepared as a control to evaluate the anti-*T. cruzi* activity. In addition, the bacterial strain was cultured with antigens of *T. cruzi* trypomastigotes (Y strain) and *Leishmania infantum* promastigotes (MHOM/BR/1972/LD), added to the Marine Agar culture medium. Two solutions of sterile seawater were prepared with the evolutionary forms of the parasites. Sterile seawater was used to induce lysis in the parasites, releasing their cellular content into the solution, followed by sonication in an ultrasonic bath. Thus, the final solution of Marine Agar medium had a parasite concentration of 2.5 × 10^6^/mL.

#### 4.5.4 Changes of Chemical Parameters

Addition to the culture medium of chemical elicitors such as EtOH (JT Baker) and DMSO (Sigma-Aldrich) at 3% (v/v) was carried out under orbital agitation at 120 rpm.

### 4.6 NMR Analysis

NMR analyses were performed on a Bruker Ascend Evo 600 spectrometer, operating at 600 MHz for the ^1^H nucleus. DMSO-d_6_ was used as the solvent and internal standard. Data were acquired and processed using MESTRENOVA software.

### 4.7 UHPLC-ESI-HR-MS/MS *-* Sample Cleanup

Samples of bacteria from solid agar incubation and from broth incubation were dissolved in 2 mL of MeOH:H_2_O (1:1, v/v) and centrifuged at 10,000 rpm at 20 °C for 15 min. The supernatant was filtered through a PTFE membrane filter (0.25 μm) and diluted to a final concentration of 1 mg/mL.

### 4.8 Mass Spectra Acquisition Using ESI-(+)-QTOF

The chromatographic separation of bacterial compounds was performed from injection of 2 μL of 1 mg/mL samples in a Shimadzu Nexera X2 UHPLC-DAD liquid chromatography system equipped with a reverse phase column (Kinetex EVO C_18_ – 2.6 μm – 100 mm × 2.1 mm, 100Å). The mobile phase applied was ultrapure H_2_O + 0.1% formic acid (A) and acetonitrile +0.1% formic acid (B), flow at 300 μL/min, at 50 °C. The chromatographic separation was conducted through a gradient method as follows: 0–2 min 5% B, 2–12 min 5–100% B, 12–15 min 100% B, 15–16 min 100–5% B, and 16–18 min 5% B to restore the initial condition. Blank samples (only MeOH) were used before and after all sample injections.

The HRMS-MS^2^ data acquisition was obtained in the positive ion mode at a mass range of *m*/*z* 75–1200 in a high-resolution spectrometer MicrOTOF-QII. The accuracy for the spectral acquisition was performed at less than 2 ppm in calibration using sodium formate. The positive ionization parameter was set as follows: the source for capillarity voltage and the end plate offset were +4500 and 500 V, respectively, dry gas (N_2_) flowing at 8.0 mL/min, pressure of 2.0 bar, and dry heater temperature of 250 °C. The auto-MS/MS was performed in CID for three precursor ions, scanning 2 × 12 Hz rate, with a fragmentation ramp using energy from 18 to 50 eV for 100–1200 Da.

### 4.9 Spectral Organization and Dereplication Using GNPS Platform

All data obtained from Bruker MicrOTOF-QII were converted in MSConvert (Proteowizard)^81^ for the centroid “.mzML”. All converted data were uploaded to the GNPS workspace through WinSCP. A molecular network was created using the online workflow (https://ccms-ucsd.github.io/GNPSDocumentation/) on the GNPS website (http://gnps.ucsd.edu).^82^ The data were filtered by removing all MS/MS fragment ions within ± 17 Da of the precursor *m*/*z*. MS/MS spectra were window filtered by choosing the top six fragment ions in the ±50 Da window throughout the spectrum. The precursor ion mass tolerance and MS/MS fragment ion tolerance were set to 0.02 Da. A network was then created using MSCluster.^83^ Edges were filtered to have a cosine score above 0.5 and more than 4 matched peaks. Edges between two nodes were kept in the network if and only if each of the nodes appeared in each other’s respective top 5 most similar nodes. The spectra in the network were then searched against GNPS’ spectral libraries. The library spectra were filtered in the same manner as for the input data. All matches kept between network spectra and library spectra were required to have a score above 0.7 and at least 4 matched peaks.

The dereplication of peptide natural product was performed using the online in-silico tool “Dereplicator (VarQuest)”^84^ based on the CMN results previously described. The parameters were set according to the QTOF high-resolution tandem mass analyzer with precursor ion mass tolerance of 0.02 Da, as well as fragment ion mass tolerance of 0.02, including the search for analogs using VarQuest. The search was performed according to the PNPdatabase with a max charge of 3, max isotopic search of 1, and minimum number of amino acids of 5, as well as max isotopic shift of 1. The advanced VarQuest options were set for the maximum allowed modification mass of 150 Da with minimum matched peaks with known compounds of 4.

The molecular networking view and editing were performed using Cytoscape v.3.9.1.^85^ Finally, all spectrum detected in the blank were removed from the networking in Cytoscape, including spectral data from the mobile phase and blank injection, as well as lone nodes with 2 spectra clustered.

### 4.10 Statistical Analysis

The EC_50_ values were determined using dose–response sigmoid curves with Graph Pad Prism 5 software. The samples were tested in duplicate and the tests were reproduced at least three times. One-way ANOVA and Tukey’s tests were used to analyze significance in the Graph Pad Prism 5 software.

## 5 Conclusions

This study demonstrated for the first time the potential of *Metabacillus indicus* to produce antitrypanosomal metabolites. The OSMAC approach, using physical alterations, proved to be the most effective strategy to improve the effectiveness of *M. indicus*, yielding metabolites with 8.4-fold higher potency. Except for chemical elicitors, the studied approaches improved the activity of the microbial metabolites against *T. cruzi*. The chemical profile of *M. indicus* in liquid and solid mediums showed a different composition in both cultivation conditions which are associated to the different EC_50_ values against *T. cruzi*. These findings confirmed that the OSMAC approach yielded not only distinct antitrypanosomal activities but also distinct metabolomic profiles in *M. indicus* that could be exploited for further drug discovery studies in Chagas disease.
